# Celastrol Suppresses Porcine Deltacoronavirus Replication by Modulating Endoplasmic Reticulum Stress‐Associated Ca^2+^ Balance

**DOI:** 10.1155/tbed/6811767

**Published:** 2026-06-28

**Authors:** Jialu Zhang, Yuqian Liu, Letao Gao, Wenzhe Liu, Ru Yan, Die Zhu, Zhouyuan Wang, Lianci Peng, Rendong Fang, Tingting Chen

**Affiliations:** ^1^ Joint International Research Laboratory of Animal Health and Animal Food Safety, College of Veterinary Medicine, Southwest University, Chongqing, 400715, China, swu.edu.cn; ^2^ Kunming Hemeihua Feed Limited Company, Kunming, 682100, Yunnan Province, China; ^3^ Department of Molecular Pharmacology, Groningen Research Institute of Pharmacy, University of Groningen, Groningen, 9713AV, Netherlands, rug.nl

**Keywords:** antiviral mechanism, calcium balance, celastrol, endoplasmic reticulum stress, porcine deltacoronavirus

## Abstract

Porcine deltacoronavirus (PDCoV) infection causes watery diarrhea and even death in neonatal piglets, leading to substantial economic losses. Meanwhile, emerging evidence indicates the potential risk of PDCoV to threaten human health and public safety. However, the effective vaccines or medicines are deficient. In this study, we evaluated the anti‐PDCoV effect of celastrol (CE), a bioactive compound extracted from *Tripterygium wilfordii*. The results showed that CE significantly inhibited PDCoV replication in a dose‐dependent manner and targeted multiple lifecycle stages of the virus. Network pharmacology analysis suggested a potential involvement of endoplasmic reticulum (ER)‐related pathways in the antiviral mechanism of CE. Further investigation demonstrated that PDCoV infection induced calcium accumulation, which was restored to the baseline by the treatment of CE. Modulation of cellular calcium using chelating or supplementation approaches also influenced PDCoV replication, indicating an association between calcium homeostasis and viral infection. In addition, PDCoV also induced ER stress and increased the calcium level associated with ER, which was attenuated by CE administration. Pharmacological inhibition of ER stress similarly reduced viral replication and altered calcium distribution. Moreover, the molecular docking analysis further suggested that CE may interact with multiple viral proteins, indicating a potential multitarget antiviral profile. Taken together, these findings demonstrate that CE inhibits PDCoV replication in LLC‐PK1 cells and is correlated with the regulation of ER stress‐associated calcium homeostasis, providing mechanistic insights into host–virus interactions during PDCoV infection.

## 1. Introduction

Coronaviruses (CoVs) are a kind of single‐stranded and positive‐sense virus with an envelope and RNA as the genetic material. The infection of CoVs has spread all over the world among various species. In the past years, CoV infection had caused death of millions of people and led to giant economic losses in the world, especially severe acute respiratory syndrome CoV (SARS‐CoV), Middle East respiratory syndrome CoV (MERS‐CoV) and SARS‐CoV‐2 [1]. Of note, these highly pathogenic CoVs are zoonotic and transmitted to humans rapidly. In the recent years, researchers also identified several novel zoonotic CoVs from patients, such as canine‐like alphacoronavirus (CCoV‐HuPn‐2018) from infants with pneumonia [2] and canine–feline–porcine‐like (CFPL) CoVs from patients with acute respiratory illness [3, 4], highlighting the potential risk of zoonotic CoVs to human health.

Porcine deltacoronavirus (PDCoV) belongs to the delta genus of CoVs with a whole genome length of 25.4 kb [5]. Different from the porcine epidemic diarrhea virus (PEDV), PDCoV mainly infects newborn piglets and causes watery diarrhea and even death. However, because of the lower mortality, PDCoV was neglected at its first emergence and has spread in the whole world so far [6]. The isolation of PDCoV from the blood of Haiti children further indicated the potential threat of PDCoV to public health [7]. However, effective medicines and strategies for the control of PDCoV are still deficient. It is crucial to discover candidate antiviral drugs against PDCoV, which also helps to identify novel antiviral targets.

Calcium exists broadly in the tissue and cells to exert effects as a regulator of signaling pathways and a messenger in cell proliferation and molecular function (MF) [8]. The main life cycle of viral infection contains entry, replication, and release, which to a large extent depends on the manipulation of cellular calcium. Human alphaherpesvirus 1 (HSV‐1) inhibited the expression of the Ca_v_3.2 T‐type Ca^2+^ channel to avoid immune detection [9]. Besides, calcium channels and pumps can be activated by viruses to facilitate viral protein biosynthesis. DENV activates the STIM1‐ORAI1 interaction to uptake more Ca^2+^ and promote the release of viral particles [10]. The endoplasmic reticulum (ER) is one of the most important organelles storing calcium and functions to maintain cellular calcium homeostasis [11]. However, the relationship between calcium ions in ER and PDCoV infection is still yet to be elucidated.

Celastrol (CE), also known as tripterine, is a pentacyclic triterpenoid compound extracted from the root, stem, and leaves of the traditional Chinese medicinal plant *Tripterygium wilfordii* [12]. Previous studies had demonstrated multiple bioactivities of CE, such as anti‐inflammation, antitumor, and cardiovascular protection [13–15]. The underlying mechanisms involve cell cycle inhibition and angiogenesis suppression mediated by the AKT/mTOR signaling pathway and cell apoptosis induction mediated by the NF‐κB signaling pathway [16, 17]. In addition, CE also exerts antiviral effects on various viruses, such as influenza A virus, hepatitis C virus, and SARS‐CoV‐2 [18–20]. However, the antiviral ability of CE against PDCoV and the underlying mechanism are still yet to be well demonstrated.

In this study, we investigated the antiviral effects of CE against PDCoV infection in vitro and explored its potential mechanisms. Our findings suggest that CE inhibits PDCoV replication and delineate the role of ER stress and calcium homeostasis in the antiviral activity of CE, providing potential targets for further therapeutics development.

## 2. Materials and Methods

### 2.1. Cell Culture

LLC‐PK1 cells (ATCC, USA), which were used for PDCoV infection and antiviral ability evaluation after infection, were maintained in Dulbecco’s modified Eagle’s medium (DMEM, Gibco, USA) supplemented with 1% penicillin–streptomycin and 10% fetal bovine serum (FBS) (Gibco, USA). Cells were incubated at 37°C with 5% CO_2_, followed by the indicated treatment.

### 2.2. Reagents

The chemicals, including CE (HY‐13067), calcium chloride anhydrous (HY‐Y0406E), and 4‐PBA (HY‐A0281), were purchased from MedChemExpress (New Jersey, USA). EGTA (pH = 7.4) (MS3583) was ordered from Shanghai Maokang Biotechnology (China). RNAiso Easy (TCH020) for total RNA extraction was ordered from Takara (Dalian, China), and TransStart one‐step gDNA removal and cDNA synthesis supermix kit (AT311‐02) and the TransStart Tip Green qPCR supermix (AQ142‐21) were ordered from TransGen Biotech (Beijing, China).

### 2.3. PDCoV Infection

PDCoV strain CHN‐HN‐1601 was donated by Professor Hanchun Yang of the China Agricultural University and stored in our laboratory. PDCoV at a final MOI of 0.1 was used for adsorption in monolayer LLC‐PK1 cells in an incubator at 37°C in 5% CO_2_ for 1 h, followed by replacement with a fresh cell culture medium consisting of DMEM supplemented with 1% trypsin. The cells were harvested for RNA isolation and protein extraction. For each condition, three individual replicates were set up.

### 2.4. Cell Viability Assay

To assess cell viability, the Cell Counting Kit‐8 (CCK‐8; C0038, Beyotime, China) was utilized following the manufacturer’s instructions. In brief, cells were first plated in 96‐well plates at optimized densities and allowed to adhere by culturing overnight under standard conditions (37°C, 5% CO_2_). Following exposure to experimental treatments, 10 µL of the CCK‐8 reagent was added to each well. The plates were then incubated for a duration ranging from 1 to 4 h. Finally, the absorbance of the samples was quantified at 450 nm employing a microplate reader.

### 2.5. Total RNA Isolation and Quantification

Total RNA of PDCoV‐infected LLC‐PK1 cells at the indicated time was extracted using an RNAiso easy reagent kit (Takara, China) according to the manufacturer’s instructions. The purity and concentration of total RNA were measured using a NanoDrop ND‐2000 spectrophotometer (Thermo Fisher Scientific, USA). The cDNA reverse‐transcribed from RNA was obtained using the TransStart one‐step gDNA removal and cDNA synthesis supermix kit (Transgen Biotech, China). Determination of the relative expression of the indicated genes was performed using the TransStart Tip Green qPCR supermix (Transgen Biotech, China) according to the comparative CT method (2^–ΔΔCT^). The reactions were performed on a CFX‐96 Real‐Time PCR System (Bio‐Rad, USA). Sequences of the forward primer and reverse primer are listed in Table 1.

**Table 1:** Primers used for qPCR.

Gene	Direction	Sequence (5′→3′)	Product size (bp)
PDCoV N	Forward	AACTTTCAGGCAGGGGCAAT	134
	Reverse	GGTTTGGTGGGTGGCTCATA	
GAPDH	Forward	ACATGGCCTCCAAGGAGTAAGA	106
	Reverse	GATCGAGTTGGGGCTGTGACT	
CASP3	Forward	TGTGGGATTGAGACGGACAG	116
	Reverse	TTTCGCCAGGAATAGTAACCAGG	
STAT3	Forward	ACATCCTTGTGTCTCCGCTG	125
	Reverse	GTATGGGGCAGCACTACCTG	
TP53	Forward	GCAGCACTAAGCGAGCACT	123
	Reverse	CTCGGAACATCTCGAAGCGT	
IL6	Forward	ATCTGGGTTCAATCAGGAGACC	126
	Reverse	ATCTGCACAGCCTCGACATT	
IFN‐β	Forward	TTCGAGGTCCCTGAGGAGATT	176
	Reverse	TCCATCTGCCCATCAAGTTCC	
MX1	Forward	GTGGAGAAAAGTCACAAAACAGGGC	288
	Reverse	TTTGCCCTTCCATTCGTCTTCT	
ISG15	Forward	GGTGAGGAACGACAAGGGTC	177
	Reverse	GGCTTGAGGTCATACTCCCC	

### 2.6. Western Blotting

Cells seeded in six‐well plates were lysed on ice with RIPA lysis buffer (Solarbio, China) supplemented with 1% phenyl methane sulfonyl fluoride (PMSF). The protein supernatants were quantified through a BCA protein concentration detection kit (Beyotime, China). Then, an equal weight of protein was separated by SDS‐PAGE and transferred onto polyvinylidene fluoride (PVDF) membranes (Millipore, USA). Membranes were blocked with 5% bovine serum albumin (BSA) in phosphate‐buffered saline with Tween‐20 (PBST) for 2 h at room temperature, followed by incubation with the indicated primary antibodies (Table 2) overnight at 4°C in a refrigerator. The next day, after three washes with PBST, membranes were incubated with correlated HRP‐conjugated secondary antibodies for 1 h at room temperature. The blots on the membrane were detected using a standard ECL detection kit (Meilunbio, China) and visualized with a Tanon 6200 imaging workstation (Tanon Science & Technology, China).

**Table 2:** Antibodies used for western blot.

Antibody	Product code	Manufacturer
PDCoV N	PDCoV11‐F	Alpha Diagnostic International
ATF6	24169‐1‐AP	Proteintech
ATF4	10835‐1‐AP	Proteintech
GAPDH	60004‐1‐Ig	Proteintech
Tubulin	80762‐1‐RR	Proteintech
HRP‐conjugated goat anti‐mouse IgG (H + L)	SA00001‐1	Proteintech
HRP‐conjugated affinipure goat anti‐rabbit IgG (H + L)	SA00001‐2	Proteintech
Orai1	ER1803‐11	HUABIO
SERCA2	ET1703‐01	Proteintech
GRP78	ER1706‐50	Proteintech
Puromycin	A21205SP	ABclonal
AF488‐labeled goat anti‐mouse IgG (H + L)	A0428	Beyotime

### 2.7. Viral Titers Detection

Cells seeded in 96‐well plates were cultured overnight until reaching 100% confluence, and then the viral liquids were serially diluted 10‐fold and inoculated into cell monolayers. After consistent incubation for several days, the cytopathic effects of cells were evaluated individually using an inverted microscope. The cytopathic wells were recorded, and the correlated viral titers were calculated by using the Reed–Muench method.

### 2.8. Immunofluorescence Staining

Cells cultivated on glass coverslips or 24‐well plates were fixed with 4% paraformaldehyde (PFA) for 30 min at room temperature. After fixation, the cells were further permeabilized in PBS supplemented with 1% Triton X‐100 (Sigma–Aldrich, USA) for 15 min. After three washes with PBS, the cells were blocked with 2% BSA for 1 h at room temperature to reduce non‐specific bindings. The cells were then incubated with anti‐PDCoV‐N antibody overnight at 4°C. The secondary antibody Alexa Fluor‐488 (Beyotime, China) was added to cells for 1 h in the dark to accomplish fluorescence staining. After three washes away from light, DAPI (Solarbio, China) was applied to stain cell nuclei. Finally, the images were captured under a Nikon fluorescence microscope.

### 2.9. Network Pharmacology Prediction

A total of 406 potential targets of CE were collected from the ITCM, BATMANTCM, Swisstargetprediction, and TCMSP databases. Meanwhile, 7332 viral diarrhea‐related targets and 8445 CoV‐related targets were retrieved from the GeneCards database using the keywords “viral diarrhea” and “coronavirus,” respectively. The intersecting genes were analyzed via an online bioinformatics platform, yielding 238 overlapping targets. The intersecting genes were uploaded into the string database, to form a protein–protein‐interaction (PPI) network, and then the network was further visualized and pictured by Cytoscape 3.9.1. In addition, these 238 common targets were submitted to the DAVID database for Gene Ontology (GO) and Kyoto Encyclopedia of Genes and Genomes (KEGG) enrichment analyses, identifying key biological processes (BPs), cellular components (CCs), MF, and critical signaling pathways underlying the antiviral activity of CE. The top GO terms and KEGG pathways were visualized as bar plots and bubble plots, respectively.

### 2.10. Calcium Measurement

Cells were seeded into a 24‐well plate and cultured overnight at 37°C. The cells were then washed three times and stained with Fluo‐4 AM. Fluo‐4 AM was diluted in a related solvent, and the cells were loaded with the working solution at 37°C for 30 min away from light. After three washes with PBS, the fluorescence images were immediately captured using a Nikon microscope.

### 2.11. ER Staining

Similar to the staining of calcium, the cells were washed twice with PBS after cultivation. Then, ER‐Tracker Red was diluted according to the protocol and incubated with cells at 37°C for 20 min in the dark. The cells were washed twice and stored in a medium without phenol red or HBSS. The images were immediately captured by using a fluorescence microscope.

### 2.12. Statistical Analysis

Data expressed in this study were expressed as mean ± standard deviation (SD) and represent the results of three independent experiments. Statistical differences were determined by one‐way ANOVAs or Student’s *t*‐tests performed using GraphPad Prism 9.0 software (GraphPad Software, CA, USA). Differences were considered to be statistically significant when the corresponding *p*‐values were <0.05, and the significances were indicated as follows:  ^∗^ *p* < 0.05;  ^∗∗^ *p* < 0.01;  ^∗∗∗^ *p* < 0.001.

## 3. Results

### 3.1. CE Inhibits PDCoV Infection in LLC‐PK1 Cells in a Dose‐Dependent Manner

**Figure 1:**
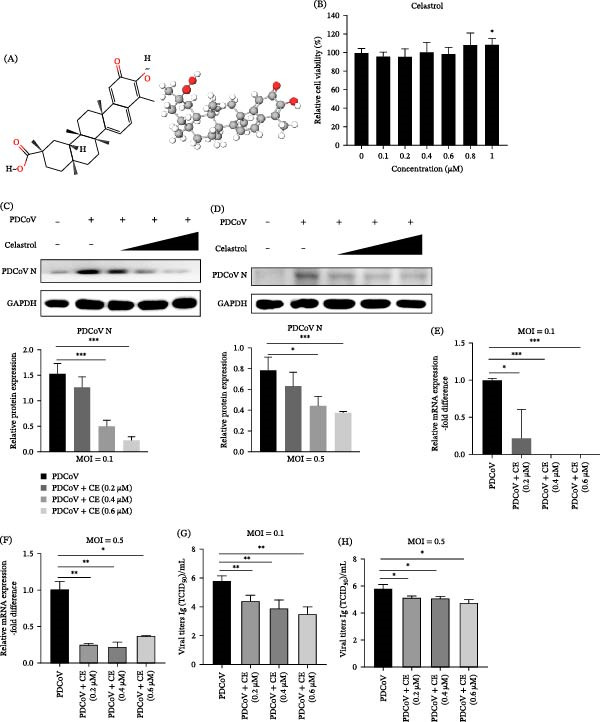
CE inhibits PDCoV infection in LLC‐PK1 cells in a dose‐dependent manner. (A) 2D and 3D structure of celastrol (CE). (B) LLC‐PK1 cells were treated with CE at the concentrations from 0 to 1 μM for 24 h. The relative cell viability was detected by using CCK‐8 assay. (C–H) The LLC‐PK1 cells were infected by PDCoV at different MOI of 0.1 and 0.5 and followed by addition of CE at the concentration of 0.2, 0.4, and 0.6 μM. (C, D) Samples were collected at 24 hpi, and PDCoV replication indicated by level of PDCoV N was detected by western blotting. The immunoblotting bands were calculated by Image J. (E, F) The transcript level of PDCoV N was measured by qRT‐PCR. (G, H) The virus titers of the cell supernatant were evaluated by TCID_50_ analysis. Data were shown as means ± SD from three independent experiments.  ^∗^ *p*  < 0.05;  ^∗∗^ *p*  < 0.01;  ^∗∗∗^ *p*  < 0.001.

The 2D chemical structure and 3D model of CE are shown in Figure 1A. To investigate the antiviral effect of CE, we first evaluate the cytotoxic effect of CE on LLC‐PK1 cells. The cells were incubated with CE at the concentrations from 0.1 to 1 μM for 24 h, and the relative cell viability was detected by the CCK8 assay (Figure 1B). The treatment of CE at a concentration of less than 1 μM showed no cytotoxic effects. Thus, nontoxic concentrations of CE (0.2, 0.4, and 0.6 μM) were chosen to determine the antiviral abilities. The protein expression levels of PDCoV N were detected by western blot, which showed that CE significantly inhibited the expression of PDCoV N after infection with different MOI in a dose‐dependent manner (Figure 1C,D). Besides, the qRT‐PCR analysis verified that the treatment of CE is effective for the inhibition of PDCoV N transcription (Figure 1E,F). Moreover, the viral titration assay further indicated the antiviral effect of CE against the progeny virus production of PDCoV (Figure 1G,H). These data demonstrate the antiviral capacity of CE against PDCoV replication in vitro.

### 3.2. CE Targets Multiple Stages of PDCoV Life Cycle

**Figure 2:**
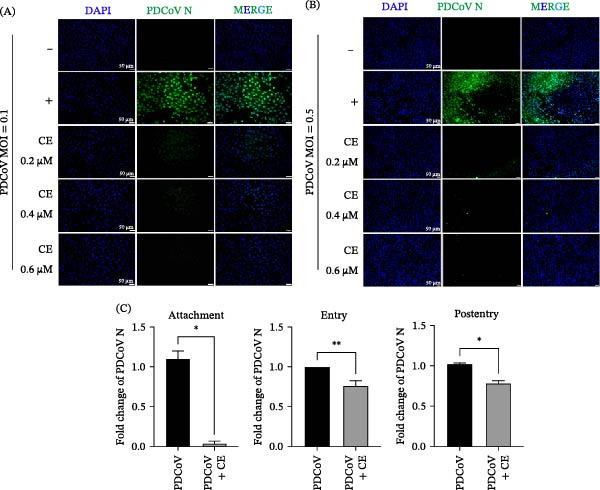
CE targets multiple stages of PDCoV life cycle. (A, B) The inhibitory effect of CE on PDCoV was further assessed by immunofluorescence staining. PDCoV N protein was stained green and the cellular nuclei were stained blue. Scale bar, 50 μm. (C) The CE was added into LLC‐PK1 cells during the PDCoV infection stages including attachment, entry, and postentry, the transcript of PDCoV N was measured by qRT‐PCR. Data were expressed as means ± SD from three independent experiments.  ^∗^ *p*  < 0.05 and  ^∗∗^ *p*  < 0.01.

The indirect fluorescence staining of PDCoV N was applied to visually evaluate the PDCoV infection after the treatment with CE. Similar to the data in Figure 1, we found that the addition of CE significantly reduces the fluorescent intensity of viral protein after infection with PDCoV at different MOIs, and 0.6 μM CE showed the best inhibitory effect (Figure 2A, B). To further confirm the concrete stage of PDCoV targeted by CE, the virus incubation conditions with LLC‐PK1 cells at different temperatures and times were divided into viral attachment (4°C for 1 h), viral entry (37°C for 1 h after viral attachment), and viral postentry (37°C for 12 h after viral entry). The qRT‐PCR analysis of PDCoV N was applied to imply the level of PDCoV. As shown in Figure 2C, the addition of CE at different stages exhibited consistently inhibitory effects on the PDCoV life cycle. These data indicate the great antiviral potential of CE.

### 3.3. Network Pharmacology Analysis of the Antiviral Activity of CE Targeting CoV‐Related Diarrhea

**Figure 3:**
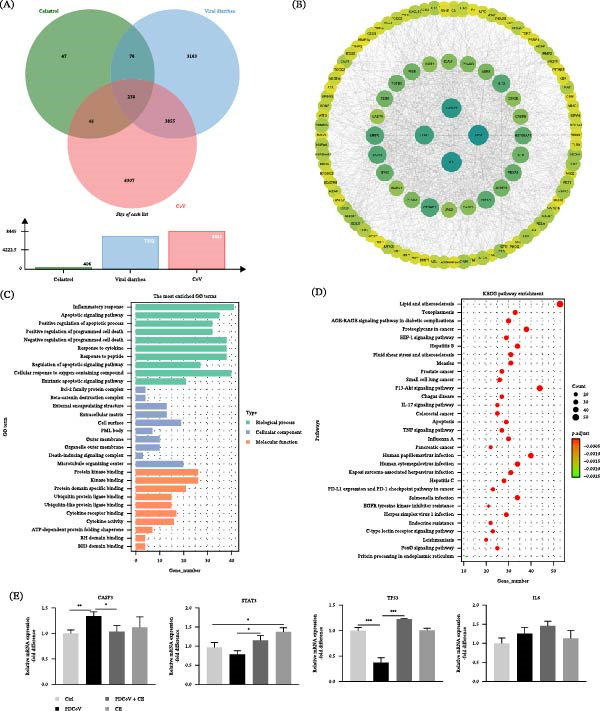
Network pharmacology analysis of the antiviral activity of CE targeting CoV‐related diarrhea. (A) CE‐, viral diarrhea‐, and CoV‐related genes were collected from indicated databases, and Venn diagram was pictured to reflect intersecting genes. (B) The network of protein–protein interaction was constructed by Cytoscape. (C, D) GO analysis and KEGG enrichment were displayed. (E) The mRNA expressions of CASP3, STAT3, TP53, and IL6 were detected by qPCR. Data were expressed as means ± SD from three independent experiments.  ^∗^ *p*  < 0.05;  ^∗∗^ *p*  < 0.01;  ^∗∗∗^ *p*  < 0.001.

In order to explore the potential mechanisms underlying the antiviral activity of CE, a network pharmacology approach was employed. A total of 238 overlapping genes were involved in the therapeutic effect of CE on CoV‐related viral diarrhea (Figure 3A). The overlapping genes were uploaded into the string database for PPI network analysis and subsequently processed and pictured by using Cytoscape. The genes were clustered by filtering different degrees of relevancy, and the top genes were CASP3, STAT3, TP53, and IL6 (Figure 3B). The GO enrichment revealed that CE mainly targeted the genes related to BP, CC, and MF; in detail, antiviral ability of CE might be related to processes including inflammatory response, apoptotic signaling pathway, positive regulation of apoptotic process, extracellular matrix, protein kinase binding, and ATP‐dependent protein folding chaperone (Figure 3C). Lipid and atherosclerosis, PI3K‐Akt signaling pathway, apoptosis, and FoxO signaling pathway were enriched in KEGG enrichment; moreover, protein processing in the ER pathway was also enriched in KEGG analysis (Figure 3D). To further verify the result of pharmacology network results, the top genes, including CASP3, STAT3, TP53, and IL6, were detected by using qPCR after the PDCoV infection and CE application. The results showed that PDCoV infection in LLC‐PK1 cells significantly enhanced the expression of CASP3 but inhibited the expression of TP53, which was reversed by the addition of CE. Moreover, the application of CE significantly raised the STAT3 expression during PDCoV infection. The IL6 expression showed an increasing tendency after CE treatment in PDCoV‐challenged cells but no significant differences (Figure 3E). These results suggest that ER‐related pathways may be involved in the antiviral effects of CE and provide a basis for further experimental investigation.

### 3.4. CE Significantly Reduces PDCoV‐Induced Calcium Accumulation

**Figure 4:**
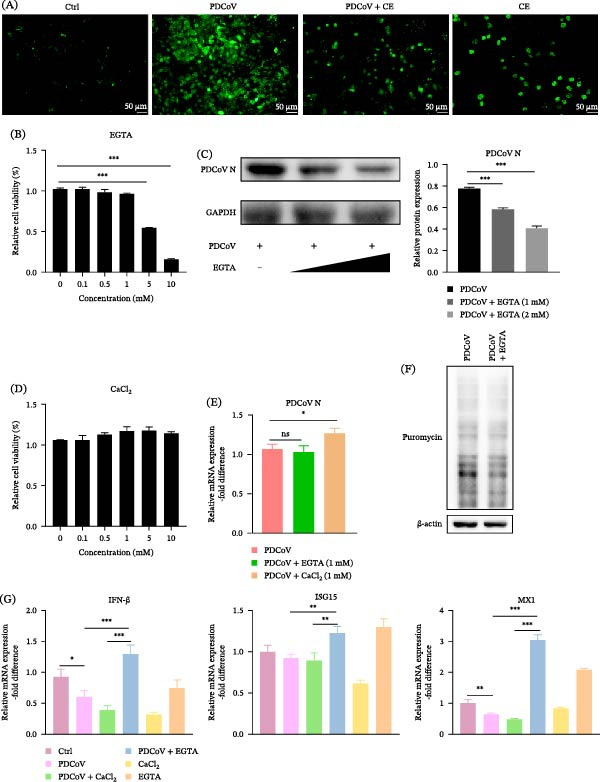
CE significantly reduces PDCoV‐induced calcium accumulation. (A) LLC‐PK1 cells were challenged by PDCoV and treated with CE at the concentration of 0.6 μM. The cells were collected at 24 hpi, and calcium ions were stained green with Fluo‐4 AM. Scale bar, 50 μm. (B) LLC‐PK1 cells were treated with EGTA at the indicated concentrations, the relative cell viability was measured by CCK‐8 analysis. (C) Cells were infected by PDCoV and followed by EGTA treatment for 24 h. The protein level of PDCoV N was analyzed by western blotting and the bands were quantified by Image J. (D) LLC‐PK1 cells were treated with CaCl_2_ at the indicated concentrations, the relative cell viability was measured by CCK‐8 analysis. (E) The transcript level of PDCoV N was measured by qRT‐PCR. (F) The protein synthesis was analyzed by treating cells with puromycin for 25 min after PDCoV infection and EGTA treatment, and then western blot analysis was applied. (G) The mRNA expression levels of genes encoding IFN‐β, ISG15, and MX1 were determined by qRT‐PCR analysis. Data were expressed as means ± SD from three independent experiments.  ^∗^ *p*  < 0.05;  ^∗∗^ *p*  < 0.01;  ^∗∗∗^ *p*  < 0.001.

Based on the enrichment of apoptosis‐related pathways and the known association between calcium signaling and apoptosis, we next examined whether calcium homeostasis is involved in PDCoV infection and CE‐mediated antiviral effects. Fluo‐4 AM staining kit was applied to detect the calcium ion level after PDCoV infection and CE treatment. The results showed that PDCoV infection induces accumulation of calcium ions, and the treatment of CE significantly reduced the calcium ions to a level approaching those of the control group at 24 hpi (Figure 4A). To further determine the effect of calcium ion level on PDCoV replication, chelating agent EGTA was used to decrease the calcium ions, whereas CaCl_2_ was used to supplement calcium ions. The CCK‐8 assay suggested that usage of EGTA at a concentration of more than 5 mM shows significant cytotoxicity (Figure 4B). Then, the protein level of PDCoV N was measured by western blot after the addition of EGTA. The treatment of EGTA showed an anti‐PDCoV effect in a dose‐dependent manner (Figure 4C). The cytotoxic concentration of CaCl_2_ was also confirmed by the CCK‐8 assay, and no cytotoxicity of CaCl_2_ was found (Figure 4D). The transcript level of PDCoV N was evaluated using qRT‐PCR after the treatment of EGTA or CaCl_2_. Of interest, EGTA treatment showed minimal effects at the transcriptional level, while the addition of CaCl_2_ at 1 mM significantly increased the expression of PDCoV N (Figure 4E). To further investigate whether the antiviral effect of EGTA correlates with the translation, we applied puromycin to monitor the translation. The results showed that the EGTA application inhibited cellular protein translation during PDCoV infection (Figure 4F). The interferon‐related genes, including IFN‐β, ISG15, and MX1, were further measured by qRT‐PCR. PDCoV infection significantly reduced the expression levels of IFN‐β and MX1. CaCl_2_ supplementation showed a trend toward reduced IFN‐related gene expression, although the changes were not statistically significant. Of note, the treatment of EGTA during the PDCoV infection significantly upregulated the expressions of IFN‐β, ISG15, and MX1 (Figure 4G). Together, these findings indicate that the alterations of calcium ion level are associated with the PDCoV replication.

### 3.5. CE Attenuates PDCoV‐Induced ER Stress and ER‐Associated Calcium Accumulation

**Figure 5:**
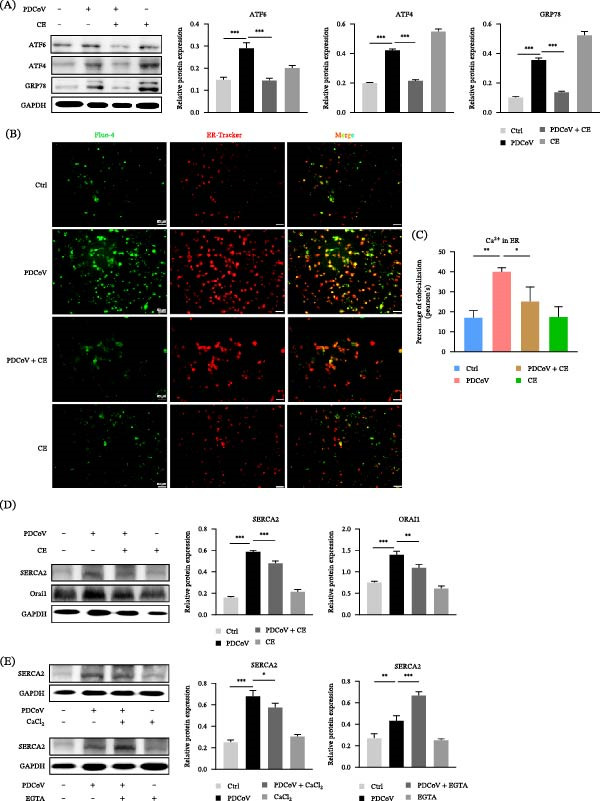
CE attenuates PDCoV‐induced ER stress and ER‐associated calcium accumulation. (A–D) LLC‐PK1 cells were infected with PDCoV (MOI = 0.1) and treated with 0.6 μM CE. The cells were processed at 24 hpi. (A) The expression levels of ER stress‐related proteins were detected by western blotting. The bands were analyzed by using Image J. (B) The cells were stained with Fluo‐4 AM and ER‐Tracker simultaneously. The calcium ions were stained green and ERs were stained red. Scale bar, 50 μm. (C) The colocalization of calcium and ER was analyzed by using Image J. (D) The expression levels of SERCA2 and Orai1 were detected by western blotting. The bands were analyzed by using Image J. (E) LLC‐PK1 cells were infected by PDCoV and treated with CaCl_2_ or EGTA for 24 h. The protein expression of SERCA2 was detected by western blotting and the bands were analyzed by using Image J. Data were expressed as means ± SD from three independent experiments.  ^∗^ *p*  < 0.05;  ^∗∗^ *p*  < 0.01;  ^∗∗∗^ *p*  < 0.001.

Given the central role of the ER in calcium storage and signaling, we next investigated whether ER stress is involved in PDCoV infection. Western blot analysis showed that PDCoV infection increased the expression of ER stress‐related proteins, including ATF4, ATF6, and GRP78, and the expression was reversed by the treatment of CE (Figure 5A). To detect the calcium ion level in ER after the infection of PDCoV, Fluo‐4 AM and ER‐Tracker were applied simultaneously, and calcium ions and ER were colocalized to evaluate the level of Ca^2+^ in ER. The results suggested that PDCoV infection induces increased colocalization of calcium ions and ER, which was shown in yellow in the Merge channel. The treatment of CE after PDCoV infection obviously reduced the amount of colocalization (Figure 5B,C). The expression of Sarcoplasmic/ER Ca^2+^ATPase 2 (SERCA2) and Orai1, proteins related to ER Ca^2+^ regulation, was further detected, which was consistent with the expression tendency of ATF6, GRP78, and ATF4. (Figure 5D). SERCA2 is an important pump protein controlling the input of calcium ions into ER; to further detect the relation between calcium ions and SERCA2 expression during the PDCoV infection, CaCl_2_ and EGTA were added separately. Similar to the data above, PDCoV infection induced significantly enhanced SERCA2 expression; nevertheless, the expression of SERCA2 was significantly declined by the addition of CaCl_2_ and was further raised by the addition of EGTA (Figure 5E). These results indicate that CE modulates ER stress and ER‐associated calcium dynamics during PDCoV infection.

### 3.6. Inhibition of ER Stress Regulates Calcium Balance to Suppress PDCoV Replication

**Figure 6:**
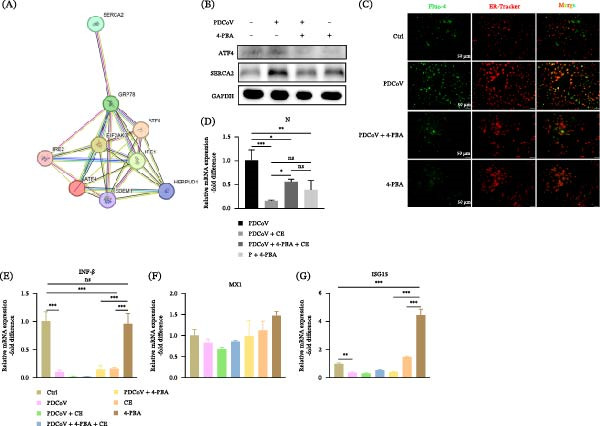
Inhibition of ER stress regulates calcium balance to suppress PDCoV replication. (A) The PPI network of ER‐stress‐related proteins and SERCA2 were pictured by STRING database. (B, C) LLC‐PK cells were infected with PDCoV (MOI = 0.1), following by the treatment of 4‐PBA. The cells were processed at 24 hpi. (B) The protein expression of ATF4 and SERCA2 was detected by western blotting. (C) The cells were stained with Fluo‐4 AM and ER‐Tracker simultaneously. The calcium ions were stained green and ERs were stained red. Scale bar, 50 μm. (D–G) LLC‐PK cells were infected with PDCoV (MOI = 0.1), following by the addition of CE and 4‐PBA individually or simultaneously. The cells were harvested at 24 hpi. The mRNA expressions of PDCoV N, IFN‐β, MX1, and ISG15 were analyzed by qRT‐PCR. Data were expressed as means ± SD from three independent experiments.  ^∗^ *p*  < 0.05;  ^∗∗^ *p*  < 0.01;  ^∗∗∗^ *p*  < 0.001.

The PPI network indicated that the ER‐related proteins interacted with SERCA2 (Figure 6A). To further investigate the antiviral mechanism of CE, 4‐PBA, an inhibitor of ER stress, was applied to confirm the role of ER stress in the regulation of calcium balance after PDCoV infection. The inhibitory effect of 4‐PBA on ER stress was confirmed by detecting the protein expressions of ATF4, and the application of 4‐PBA significantly downregulated the expression of ATF4 and SERCA2 during PDCoV infection (Figure 6B). Besides, the addition of 4‐PBA reduced the calcium level in the ER after PDCoV infection, indicated by the colocalization of calcium and ER (Figure 6C). PDCoV replication was suppressed by 4‐PBA, indicated by the expression of PDCoV N (Figure 6D). The PDCoV‐suppressed immune response was indicated by the downregulating expressions of IFN‐β and ISG15. Of note, the sole application of 4‐PBA significantly increased the expression of ISG15 but failed to affect IFN‐β and MX1. Moreover, the addition of CE or 4‐PBA during the PDCoV infection showed no effect on the expressions of IFN‐β, MX1, and ISG15 (Figure 6E–G). These findings suggest that inhibition of ER stress is associated with reduced PDCoV replication and altered calcium homeostasis.

### 3.7. In Silico Prediction of CE Binding to Viral Proteins

**Figure 7:**
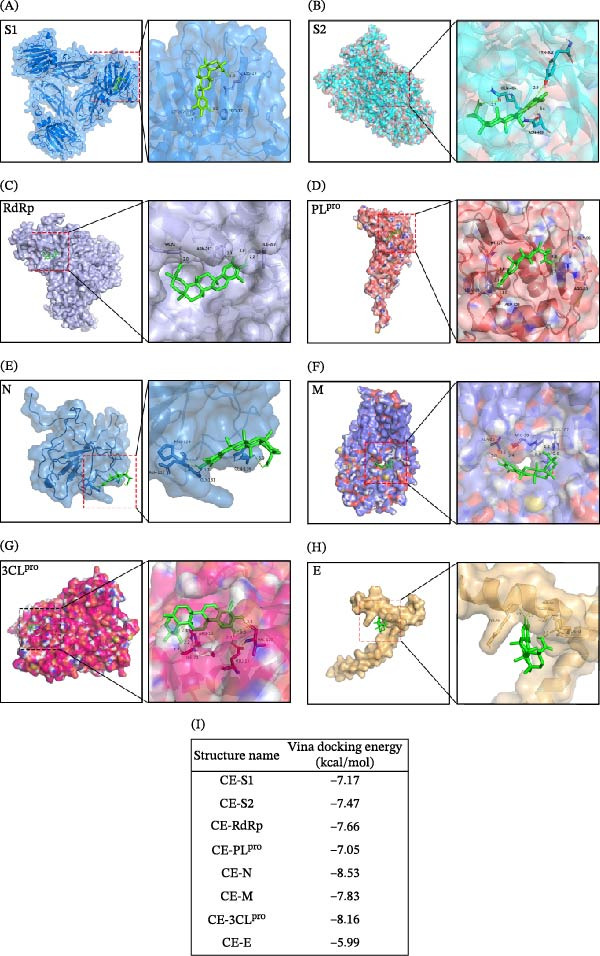
CE shows potential binding affinity to viral proteins. (A, B) Molecular docking results of CE and S1 subunit or S2 subunit of PDCoV S protein. (C) Molecular docking results of CE and PDCoV RNA‐dependent RNA polymerase (RdRp). (D) Molecular docking results of CE and PDCoV papain‐like protease (PL^pro^). (E) Molecular docking results of CE and PDCoV N protein. (F) Molecular docking results of CE and PDCoV M protein. (G) Molecular docking results of CE and PDCoV 3‐chymotrypsin‐like protease (3CL^pro^). (H) Molecular docking results of CE and SARS‐CoV E protein (ID: 2MM4). (I) The vina docking energy for the structures are as shown in the table.

Considering the calcium‐regulating ability of CE investigated above and the key roles of calcium in multiple cellular processes, we analyzed the binding activity of CE with the key viral proteins during the viral life cycle by using Autodock 4. As is shown in Figure 7, the proteins evaluated included S1, S2, RdRp, PL^pro^, N, M, and 3CL^pro^ of PDCoV, and E (ID: 2MM4) of SARS‐related CoV. Molecular docking analysis predicted that CE may interact with these proteins and exhibited favorable binding energies ranging from −5.99 to −8.53 kcal/mol (Figure 7I). These findings indicate that CE has potential binding affinity to multiple viral proteins, suggesting a possible multitarget antiviral profile.

## 4. Discussion

Nowadays, the outbreak of COVID‐19 hinted at the potential risk of “X disease,” which was also predicted as a “SARS‐CoV‐related virus” by the World Health Organization [21]. As one of the most widely spreading porcine CoVs in the world, PDCoV not only causes great loss to the pig industry but also threatens food security and human health. Because of the smallest genome of PDCoV in CoVs, it raised the concern that PDCoV might mutate to more virulent strains and the transmission of PDCoV among species [22]. Thus, it is crucial to investigate antiviral agents for the control of PDCoV and unpredicted diseases in the future.

The management of treating COVID‐19 also implies the key role of repurposing existing drugs. The antitumor and antiinflammation effects of CE have been proved by multiple studies in the past years, indicating its therapeutic potential [23, 24]. However, it was rarely explored and applied in the treatment of infectious diseases. In this study, we demonstrated the excellent antiviral ability of CE on PDCoV, which showed similar antiviral capacity in infection with different MOI. In addition, CE affected multiple stages of the viral life cycle to impede PDCoV replication, especially the attachment stage, which is consistent with a previous study indicating the anti‐SARS‐CoV‐2 effect by binding to S protein [25], supporting further investigation of CE as a candidate antiviral compound.

To further specify the potential antiviral mechanism of CE, we applied network pharmacology analysis targeting CoV‐related diarrhea. The results mainly concentrated on the genes related to apoptosis, the upregulated CASP3 expression induced by PDCoV infection was inhibited by CE treatment, which indicates the pharmacological effect of CE on apoptosis and is similar to the previous analysis targeting PEDV infection [26]. In addition to apoptosis, we noticed that the ATP‐dependent protein folding chaperone and protein processing in ER were also enriched, indicating the important role of ER in the antiviral mechanism of CE. In the top hub proteins, except for the apoptotic gene and immune‐related genes, TP53 was also screened. In the previous study, TP53 was recognized with an important role in immunity and antiviral defense [27], which was significantly suppressed in PDCoV‐infected cells, consistent with the immune‐escaping characteristic of PDCoV. In earlier studies, TP53 in the ER was shown to regulate the balance of calcium under the stimulation of stress [28]. Cellular calcium concentration was also proven to participate in the regulation of apoptosis; besides, it is well known that calcium change triggers various cellular functions [29]. The homeostasis of calcium also affected the viral infection; for example, the Ca^2+^ influx was targeted by verapamil HCl to suppress PRRSV infection [30], and ORF3 of PEDV was recognized as a viroporin to facilitate PEDV replication [31]. Ca^2+^ also showed regulatory effects on pattern recognition receptor (PRR) response. For instance, intracellular Ca^2+^ activates innate immune responses via the stimulation of PRR and IRF 3/7 [32]. In this study, we observed that PDCoV infection induced intracellular calcium accumulation, which was attenuated by CE treatment. Furthermore, modulation of calcium levels using EGTA or CaCl_2_ influenced PDCoV replication, suggesting that calcium homeostasis affects viral infection. Interestingly, the different effects of calcium modulation on viral mRNA and protein levels may indicate the involvement of posttranscriptional regulatory mechanisms. The puromycin application proved that it could be used as a translation monitor by being incorporated into newly synthesized polypeptides [33]. The downregulated puromycin level after EGTA treatment in PDCoV‐infected cells might indicate the important role of the cellular calcium level in PDCoV replication. Moreover, IFN‐related genes were also affected by the chelation of Ca^2+^, which might participate in the anti‐PDCoV effect of EGTA.

ER is an important site for energy exchange and protein expression, which also participates in the Ca^2+^ regulation. Previous studies showed that various viral infections induce ER stress and unfolded protein reaction (UPR) with different degrees, including SARS‐CoV‐2 and PRRSV [34, 35]. PDCoV also possesses the ability to induce ER stress and UPR; besides, the earlier research also indicated that the PERK signaling pathway negatively regulates PDCoV replication, while the ATF6 signaling shows a positive regulatory effect [36]. Our data showed that PDCoV infection induces cellular ER stress indicated by upregulated ATF4, ATF6, and GRP78 expressions and enhanced ER‐associated calcium accumulation, which were reversed by the application of CE, suggesting that CE may modulate ER stress and calcium balance to impede PDCoV replication.

SERCA2, a key calcium pump responsible for transporting Ca^2+^ into the ER, interacts with the envelope protein of SARS‐CoV‐2 via the formation of oligomers with regulins, thus resulting in the reduction of ER Ca^2+^ reload [37]. However, the induction of SERCA2 expression was indicated in the PDCoV infection, which was suppressed by the addition of CE. However, the application of CaCl_2_ and EGTA showed a reverse tendency contrary to the hypothesis, which might reflect compensatory cellular responses to altered calcium levels. The ER chaperone‐like GRP78 not only activates UPR under ER stress but also accounts for the ER calcium buffering [38]. In the diabetic model, continuous UPR activation under the condition of high glucose leads to ER Ca^2+^‐dependent apoptosis [39]. However, the regulatory effect of ER stress‐induced UPR on the ER calcium level was not well elucidated in PDCoV infection. Inhibition of ER stress by 4‐PBA was associated with reduced viral replication and decreased ER‐associated calcium levels. These findings indicate that ER stress and ER‐related calcium homeostasis are closely associated with PDCoV replication. CE attenuated ER stress and calcium accumulation induced by PDCoV, suggesting that modulation of ER stress‐associated calcium homeostasis may contribute to its antiviral ability.

In addition, considering the extremely low antiviral concentration in this study, we evaluated the binding activity of CE with several proteins from PDCoV. These computational predictions suggest several potential viral targets of CE that require experimental validation in future studies.

However, several limitations of this study should be acknowledged. The mechanisms demonstrated in this study provide new insights to understand the antiviral action of CE, but the precise mechanisms still require further investigation to be fully elucidated. Moreover, the antiviral potential was evaluated by an in‐vitro study; in vivo validation is required to confirm the antiviral efficacy of CE.

## 5. Conclusion

In conclusion, our study demonstrates that CE inhibits PDCoV replication in LLC‐PK1 cells and is associated with the modulation of ER stress and calcium homeostasis. These findings provide new insights into host–virus interactions and suggest that targeting ER‐associated calcium signaling pathways may represent a potential strategy for controlling PDCoV and other emerging viruses. Further studies in animal models are required to validate these observations.

## Author Contributions

**Jialu Zhang:** writing – original draft, writing – review and editing, software, methodology, formal analysis, conceptualization, funding acquisition. **Yuqian Liu:** writing – review and editing, methodology, formal analysis. **Letao Gao** **, Wenzhe Liu, Ru Yan, and Die Zhu:** methodology, formal analysis. **Zhouyuan Wang:** resource. **Lianci Peng:** software, methodology. **Rendong Fang:** writing – review and editing, funding acquisition. **Tingting Chen:** writing – review and editing, visualization, supervision.

## Funding

This work was supported by the National Natural Science Foundation of China (Grants 32503119 and 32473027), the Fundamental Research Funds for the Central Universities (Grant SWU‐KQ25021), the Natural Science Foundation of Chongqing, China (Grant CSTB2025NSCQ‐GPX0489), the Chongqing Modern Agricultural Industry Technology System (Grant CQMAITS202512), the Yunnan Province Science and Technology Talents and Platform Program (Grant 202405AF140106), and the Co‐construction Project of Fuling Academy of Southwest University (Grant FLYJY202506).

## Ethics Statement

This study does not require ethical approval because no animals or humans were involved.

## Conflicts of Interest

The authors declare no conflicts of interest.

## Data Availability

The data that support the findings of this study are available from the corresponding author upon reasonable request.
